# Fast-Growing Magnetic Wood Synthesis by an In-Situ Method

**DOI:** 10.3390/polym14112137

**Published:** 2022-05-24

**Authors:** Istie Rahayu, Esti Prihatini, Rohmat Ismail, Wayan Darmawan, Lina Karlinasari, Gilang Dwi Laksono

**Affiliations:** 1 Department of Forest Products, Faculty of Forestry and Environment, IPB University, Bogor 16680, Indonesia; esti@apps.ipb.ac.id (E.P.); wayandar@indo.net.id (W.D.); karlinasari@apps.ipb.ac.id (L.K.); gilanglksno@gmail.com (G.D.L.); 2 Department of Chemical, Faculty of Mathematics and Natural Sciences, IPB University, Bogor 16680, Indonesia; rohmatchemistry@apps.ipb.ac.id

**Keywords:** impregnation, jabon, magnetic wood, physical properties

## Abstract

Jabon (*Anthocephalus cadamba*) is a fast-growing wood with low quality due to its low density and strength. The quality can be increased by modifying the wood through impregnation with various chemical compounds. In this study, jabon was impregnated with a solution of Fe and immersed in a strong base (NaOH) or a weak base (NH_4_OH) to form magnetite (Fe_3_O_4_) in-situ. This study analysed the use of NaOH and NH_4_OH in synthesising magnetic jabon wood and evaluated the wood’s characteristics. The impregnation process began with a vacuum of −0.5 bar for 0.5 h and then a pressure of 1 bar for 2 h. The samples subsequently underwent assessment of their dimensional stability, density, and characteristics. The results showed that impregnation with Fe solution followed by NaOH or NH_4_OH significantly affected the density and dimensional stability of the wood. The polymer weight gain was higher with NaOH, while the anti-swelling efficiency was higher with NH_4_OH. The density and bulking effect were increased, but the water uptake was decreased. Fourier transform infrared analysis showed the successful synthesis of magnetite. Scanning electron microscopy–energy-dispersive X-ray spectroscopy analysis revealed that magnetite covered the vessel fibre cell walls, and vibrating sample magnetometry analysis showed significant magnetic properties of the wood.

## 1. Introduction

Based on data from the Indonesian Ministry of Environment and Forestry [1], the wood supply from plantation and community forests in Indonesia increased from 37.3 million m^3^ in 2015 to 46.6 million m^3^ in 2018; meanwhile, the wood supply from natural forests decreased from 8.3 million m^3^ [2] to 5.7 million m^3^ [1,3], over the same time period. It is evident that community forests play an important and growing role in the country’s wood supplies. Jabon (*Anthocephalus cadamba*) is widely planted in community forests in Indonesia; in 2019, more than 9 000 m^3^ of jabon was harvested in Indonesia [4]. The species is considered native not only to Indonesia, but also to India, Bangladesh, Cambodia, China, Laos, Myanmar, New Guinea, Sri Lanka, Thailand, and Vietnam, and Jabon has also been introduced in Guatemala and El Salvador [5]. Jabon is harvested at 5–7 years old, an age at which the wood has inferior physical and mechanical properties and low durability; however, wood modification can improve its qualities and expand its applications. One wood modification method is impregnation with chemical compounds. Considerable research has been conducted on the impregnation of fast-growing wood using methyl methacrylate [6,7,8] and phenol-formaldehyde [9,10]. Nevertheless, the use of fast-growing wood species, especially jabon, is still limited for carpentry and packaging. Jabon wood is generally used for packaging in Indonesia, but special treatments could add value by improving properties or allowing new functionalities and thereby expanding its applications. In this study, we aimed to make jabon wood magnetic, which would enhance its properties and thus allow it to serve new functions and have broader applications.

Magnetic wood is essentially a combination of wood with magnetic powder or magnetic fluid. Iron oxide nanoparticles, such as magnetite (Fe_3_O_4_) and maghemite (g-Fe_2_O_3_), are widely used as non-ferrofluid materials, with the former being preferred owing to greater saturation magnetisation [11]. Magnetic wood has advantages, such as increased dimensional stability of wood, magnetic properties that can attract or be attracted to magnets, and absorbance of electromagnetic waves [12,13]; thus, magnetic wood has broad new functions and uses, and it can be applied as a construction material because it is able to withstand heat [14]. In the future, magnetic jabon wood could become a superparamagnetism material. Such materials can be used in energy harvesting, heat transfer, and the adsorption and removal of contaminants such as heavy metals, dyes, and organic compounds [15].

Magnetic wood manufacture can be done through in-situ methods [16,17,18] and hydrothermal methods [19,20] among others. In this study, we utilized the practical and simple in-situ method, so that we could make jabon magnetic wood according to our own shapes and dimensions. The in-situ method consisted of impregnation with a mixture of Fe^2+^ and Fe^3+^, followed by immersion in either a strong base sodium hydroxide (NaOH) or a weak base ammonium hydroxide (NH_4_OH) as a precursor [21]. The result of this process was the formation of Fe_3_O_4_ in the wood; this in-situ method was carried out previously by researchers using ammonium hydroxide (NH_4_OH) in poplar [21]. According to Zhang et al. [22], the size of the Fe_3_O_4_ nanoparticles formed in the wood is larger when NH_4_OH is used as the precipitator compared with NaOH. The differences in size are due to variations in the process of nucleation and grain growth during synthesis. Because NH_4_OH is a weak base, it produces a smaller number of magnetite cores, which favours crystal growth and Ostwald ripening, resulting in particles with larger sizes. When NaOH, a strong base, is used, the formation of a greater number of magnetite cores occurs [23]. Based on these previous studies, we compared the utilisation of NaOH and NH_4_OH as precursors in this study. Our objectives were to analyse the effectiveness of NaOH and NH_4_OH as precursors for binding Fe to jabon wood and to characterise the resultant magnetic jabon wood.

## 2. Materials and Methods

Samples of 5-year-old jabon wood that was free of defects were obtained from a community forest in the Bogor area, West Java, Indonesia. The jabon log was then cut into 100-cm-long sections. All samples came from the same tree and were cut at the same time to get uniform wood samples. The chemicals used in this study included FeCl_3_∙6H_2_O, FeCl_2_∙4H_2_O, NaOH, and NH_4_OH, and additional supplies included pH paper and deionized water. The equipment used included a chain saw, table circular saw, analytical balance, callipers, oven, fan, vacuum Erlenmeyer tube, impregnation tube, magnetic stirrer, and moisture meter. The study also used instruments for ultrasonication, scanning electron microscopy (SEM) and energy-dispersive X-ray spectroscopy (EDX) analysis, Fourier transform infrared spectrometry (FTIR), X-ray diffraction (XRD) analysis, and vibrating sample magnetometry (VSM).

### 2.1. Preparation of Wood Samples

Jabon wood was cut using a chain saw and a circular saw table, without distinguishing between sapwood and heartwood. Each test sample was 2 cm × 2 cm × 2 cm [24]. A total of 30 samples were used in 10 replications of each experimental condition: no treatment, in-situ NH_4_OH, and in-situ NaOH. The samples were used in testing weight polymer gain (WPG), anti-swelling efficiency (ASE), water uptake (WU), bulking effect (BE), and density.

### 2.2. Preparation of Impregnation Solutions

The impregnation solution contained a mixture of Fe^2+^ and Fe^3+^. The compounds FeCl_3_∙6H_2_O and FeCl_2_∙4H_2_O were prepared according to the calculation of the mole ratio of Fe^2+^:Fe^3+^ = 1:1.6 [21] in a 100-mL beaker. The two compounds were then put into a 250-mL beaker and 100 mL of deionized water was added. The Fe solution was stirred for 15 min until homogeneous, using a magnetic stirrer on a scale of 3–4, while in a closed/airtight state to avoid material contamination and metal oxidation reactions.

### 2.3. The Impregnation Process

The sample was first oven-dried at a temperature of 103 ± 2 °C until the weight was constant. Then, the sample was weighed and measured before impregnation. The impregnation solution was then poured into 10 containers that each held a wood sample, with a volume of 10 mL each. The bottles of the film were then inserted into the impregnation tube. The impregnation process began with a vacuum of −0.5 bar for 0.5 h and a pressure of 1 bar for 2 h. After the impregnation process was completed, the remaining Fe mixed solution was removed. The samples then underwent an immersion process for 24 h. The immersion solution was either a strong base, 4 M NaOH [25], or a weak base, 25% NH_4_OH. After the immersion was completed, the wood was rinsed with deionized water to remove the alkaline residue and then wrapped in aluminium foil and heated at 65 °C for 12 h. Finally, it was dried at 105 °C for 2 days (until constant weight). Weighing and measuring the wood samples were carried out.

Calculation of the WPG was calculated by the following formula:

WPG (%) = [(W_1_ − W_0_)/W_0_] × 100 (1)

where W_0_ is the initial oven-dried weight of the sample before impregnation, and W_1_ is the oven-dried weight of the sample after impregnation.

ASE testing was carried out by means of repeated water immersion [26]. ASE was calculated by the following formula:

ASE (%) = [(S_u_ − S_t_)/S_u_] × 100 (2)

where S_u_ is the volume shrinkage of the untreated wood sample, and S_t_ is the volume shrinkage of the treated wood.

WU testing on samples was carried out after immersion in water for 24 h. WU was calculated by the following formula:

WU (%) = [(W_2_ − W_1_)/W_1_] × 100 (3)

where W_1_ is the sample weight after impregnation treatment, and W_2_ is the sample weight after immersion in water for 24 h.

The BE test was calculated by the following formula:

BE (%) = (V_1_ − V_0_)/V_0_ × 100 (4)

where V_0_ is the initial oven-dried volume of the wood sample before impregnation and V_1_ is the oven-dried volume of the wood sample after impregnation.

Oven-dried density (ρ) was calculated by considering WPG and BE values and oven-dried density of untreated samples at baseline (Formula (5)).

(5) $$\rho \;(g/{\mathrm{cm}}^{3})=\frac{B}{V}\times \;100$$

where B is the weight of the sample before or after impregnation treatment, and V is the volume of the sample before or after impregnation treatment.

### 2.4. Characterisation of Impregnated Jabon Wood

#### 2.4.1. Fourier Transform Infrared Spectrometry

Untreated and treated wood samples were milled into 200-mesh particle and embedded in potassium bromide (KBr) pellets. The finished pellets were then analysed by FTIR (Perkin-Elmer Spectrum One) and scanned at a wave number range of 4000 to 400 cm^−1^ with a resolution of 4 cm^−1^ for 32 scans.

#### 2.4.2. Scanning Electron Microscopy and Energy-Dispersive X-ray Spectroscopy

The penetration and distribution of nano Fe_3_O_4_ in wood cell walls were analysed using JEOL Brand SEM (JSM-6510LA series). The untreated and treated wood samples were cut to a size of 0.5 cm × 0.5 cm × 0.5 cm on a tangential plane, placed on conductor adhesive, coated with gold, and observed under SEM at a voltage of 20 kV. Wood samples also underwent EDX analysis to determine the magnetic iron content within the treated wood.

#### 2.4.3. X-ray Diffraction Analysis

Wood samples were cut into 2 mm thick pieces in a tangential direction. The degree of crystallinity of the wood sample (incision) was analysed by using an XRD-PANAnalytical Empyrean type with a 1D PIXcel detector. The parameters used in this device were Cu Kα radiation with a graphite monochromator, a voltage of 40 kV, a current of 30 mA, and a scan range of 2θ between 5° and 80° for the degree of crystallinity and between 5° and 90° for phase analysis. The scanning speed used was 2°/min.

#### 2.4.4. Vibrating Sample Magnetometric

VSM testing was carried out using VSM type Oxford VSMl.2H with Oxford Object Bench software; this VSM uses a horizontal electromagnet type external field system with a working area from −1 T to 1 T and a maximum field change rate of 50 G/s. The voltage measurement area was 0.1–1000 mV, the sample rotation was 720° with a vibration frequency of 55 Hz. In addition, the saturation magnetisation (M_s_), coercivity (H_c_), and retentivity (M_r_) were also evaluated. To assess the stability of magnetic properties in an acidic environment, an acid resistance test was carried out by immersing the specimen in a 4% hydrochloric acid solution for 7 days. Then, its magnetic properties were evaluated by VSM. The dimensions of the specimen for the magnetic test were 3 mm × 3 mm × 1.5 mm in the longitudinal direction.

### 2.5. Data Analysis

This study used a completely randomized design and data were evaluated using analysis of variance (ANOVA), followed by Duncan’s test at a significance level of = 1%. Statistical testing was done using the IBM SPSS Statistics (Statistical Package for Service Solutions) version 25.0 program Stanford, California, CA, United States.

## 3. Results

### 3.1. Dimensional Stability of Magnetic Jabon Wood

The results of the dimensional stability (WPG, ASE, BE, and density) of magnetic jabon wood are presented in Table 1. In general, the in-situ method was successful in increasing all dimensional stability parameters. The research results of Dong et al. [21] also showed the same trend in poplar wood.

The WPG values (Table 1) of magnetic jabon wood treated by a strong base (NaOH) or a weak base (NH_4_OH) used as the precursor were 47.4% and 31.4%, respectively. The WPG value associated with NaOH was higher than that associated with NH_4_OH. This result revealed that the amount of magnetite (Fe_3_O_4_) produced by NaOH as the precursor was higher than that produced by NH_4_OH, and can be explained by the reaction shown in Equation (6) for the formation of magnetite [25]:

2Fe^3+^(aq) + Fe^2+^(aq) + 8OH^−^(aq) → Fe_3_O_4_(s) + 4H_2_O (6)

Wood density is the ratio between the weight and the volume of wood. As shown in Table 1, the treated wood had a higher density value than the untreated wood in the current study. Based on statistical tests, the difference in wood density values was not significant (Table 1); this result is in line with Merk et al. [17], who found that magnetic Norway Spruce and European Beech retained their natural wood properties after treatment (low-density magnetic wood). The in-situ treatment did not degrade the wood structure. As the WPG value increases, the wood density should also increase; however, the increase in the WPG value was not accompanied by an increase in ASE, and the WU decreased. The higher the ASE value, the lower the ability of jabon wood to adsorb water from the surrounding environment. In addition, the lower the WU value, the less water can enter the magnetic jabon wood.

### 3.2. Magnetic Jabon Wood Characterisation

The characteristics of magnetic jabon wood were characterised by using FTIR analysis, SEM-EDX, XRD analysis, and VSM.

#### 3.2.1. FTIR Analysis

**Figure 1:**
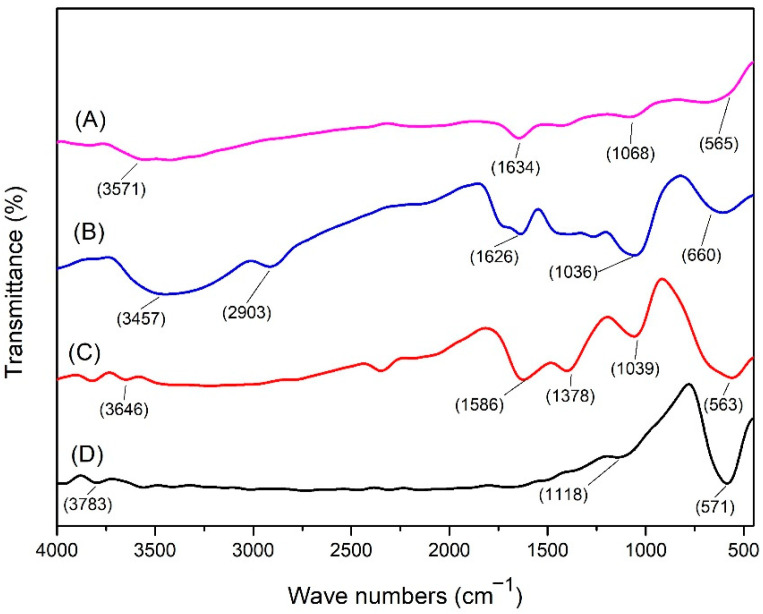
FTIR spectrum for (**A**) magnetic jabon wood from the in-situ method with NH_4_OH; (**B**) untreated jabon wood; (**C**) magnetic jabon wood from the in-situ method with NaOH, and (**D**) the magnetic standard.

The results of the identification of functional groups in commercial magnetite powder (nano Fe) (Figure 1) show that at a wave number of 571 cm^−1^ there is a functional group Fe(III)-O (stretching vibration), and at a wave number of 1118 cm^−1^ a functional group appears. OH (OH out-of-plane bending) and at a wave number of 3783 cm^−1^ there is an -OH (bending vibration) group [27,28].

From the FTIR analysis of untreated wood, a CO functional group appeared at wave number 1036 cm^−1^, CH functional group of lignin at wave number 660 cm^−1^, functional group C=C at wave number 1626 cm^−1^, CH functional group of cellulose and hemicellulose at wave number 2903 cm^−1^, and an OH functional group at a wave number of 3457 cm^−1^ [28]. The functional groups identified in magnetic wood following in-situ treatment with NaOH and NH_4_OH precursors showed slight differences from those in the untreated samples, particularly with regard to the appearance of the Fe-O (stretching vibration) functional group, which represents a bond in the magnetite compound (F_3_O_4_). In the in-situ treatment group with a strong base precursor, the Fe-O (stretching vibration) functional group appeared at a wave number of 563 cm^−1^, while in the treatment group with a weak base precursor, it formed at a wave number of 565 cm^−1^. The FTIR analysis also revealed that the C-H bending aromatic group was not identified from lignin in the magnetic wood [29]; this result could be caused by the breaking of bonds in the lignin structure due to the base immersion treatment [30].

#### 3.2.2. Macro- and Microscopic Analysis (SEM and SEM-EDX Analysis)

**Figure 2:**
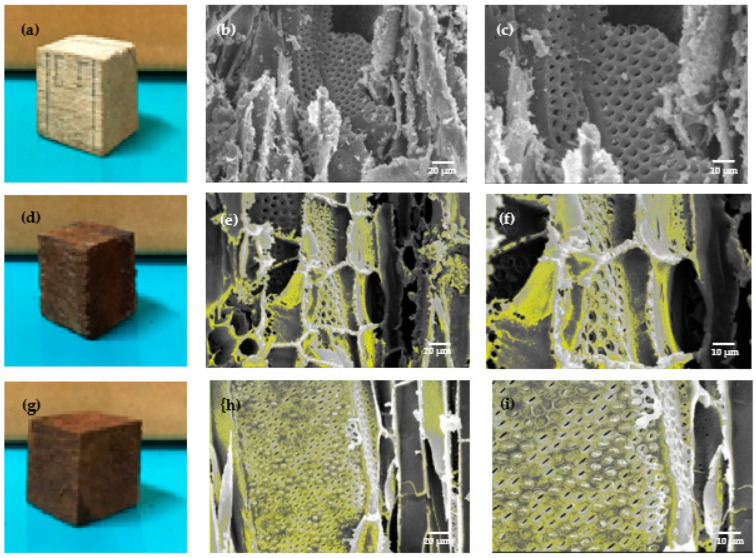
(**a**) Untreated jabon wood; (**b**) tangential section of untreated jabon wood at ×550 magnification; (**c**) tangential section of untreated jabon wood at ×1000 magnification; (**d**) magnetic jabon wood after in-situ treatment with NaOH precursor; (**e**) tangential section of magnetic jabon wood after in-situ treatment with NaOH at ×550 magnification; (**f**) tangential section of magnetic jabon wood after in-situ treatment with NaOH at ×1000 magnification; (**g**) magnetic jabon wood after in-situ treatment with NH_4_OH; (**h**) tangential section of magnetic jabon wood after in-situ treatment with NH_4_OH at ×550 magnification, and (**i**) tangential section of magnetic jabon wood after in-situ treatment with NH_4_OH at ×1000 magnification.

The Fe_3_O_4_ impregnated wood appeared brown in colour compared with the untreated wood (Figure 2a,d,g). Jabon wood showed a darker colour on its cell walls after impregnation due to the formation of black Fe_3_O_4_ (Figure 2f,h,i); this result aligned with that of Merk et al. [17], who reported dark brown colour on treated samples, and magnetite deposit along cell walls of treated spruce and beech samples.

Figure 2 shows the notable differences between untreated and treated jabon wood. Apart from magnetite covering the cell walls, deposits were also present in the cell cavities (the yellow colour in Figure 2e,f,h,i depicts magnetite). The yellow colour of the magnetic jabon wood for which a strong base was used in the in-situ treatment (Figure 2e,f) is darker than that of the wood for which the weak base precursor was used (Figure 2h,i); this indicates that more magnetite was formed when we used the strong base precursor. In-situ synthesized jabon wood with either a strong or weak base precursor was mostly covered by iron particles, which can be seen in the SEM-EDX results (Table 2).

Based on the results of quantitative analysis using SEM-EDX results showed that the Fe content in magnetic jabon wood had a higher value when the wood was treated with a strong base precursor (40.11%) than with a weak base precursor (23.39%).

#### 3.2.3. XRD Analysis

**Figure 3:**
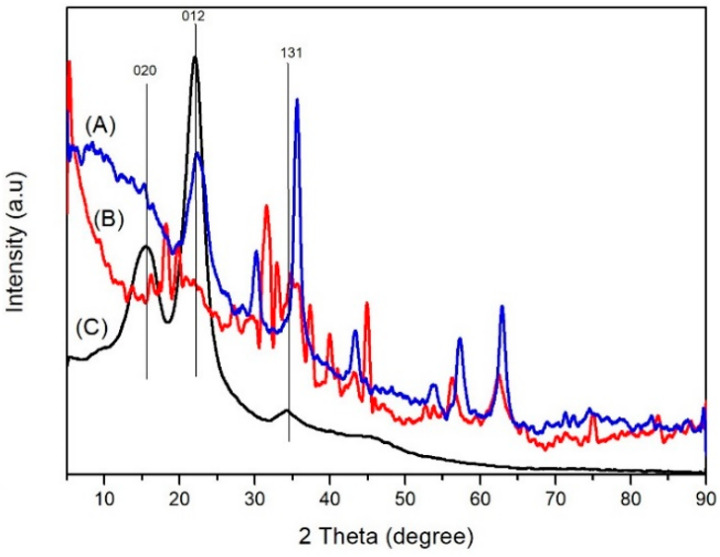
Diffractogram from XRD analysis of (**A**) of magnetic jabon wood treated in-situ with NaOH precursor (blue line); (**B**) magnetic jabon wood with NH_4_OH precursor (red line), and (**C**) untreated jabon wood (black line).

**Figure 4:**
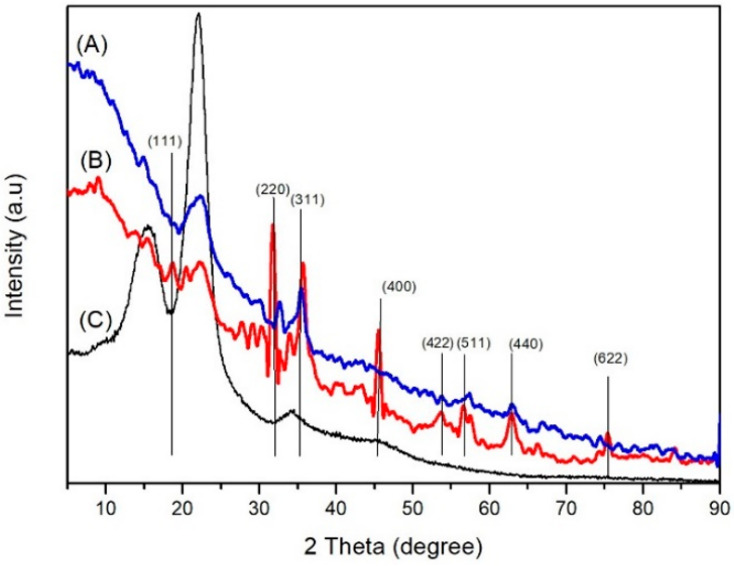
Diffractogram XRD analysis of (**A**) magnetic jabon wood after in-situ treatment with NaOH precursor (blue line); (**B**) magnetic jabon wood after treatment with NH_4_OH precursor (red line), and (**C**) untreated jabon wood (black line).

The XRD analysis (Figure 3C) of the untreated wood showed peaks at values 2θ 15, 30, 21.97, and 34.1, which indicate the crystal planes (020), (012), and (131) of cellulose. In the diffractogram of the magnetic jabon wood created with a strong base precursor (Figure 4A), there are peaks at three values, namely 16.24, 20.9, and 34.61. In the diffractogram of the magnetic jabon wood created with a weak base precursor (Figure 4B), there are peaks at two values, namely, 15.25 and 22.48. XRD analysis showed that the magnetite compound had been formed in-situ in the jabon wood, with the use of both strong and weak bases as precursors. In the diffractogram (Figure 4A), magnetic jabon wood made with a strong base precursor has peaks at a value of 2θ, namely 32.57, 35.41, 53.67, 57.39, 62.89, and 74.21. In the diffractogram of the magnetic jabon wood made with weak base precursors (Figure 4B), there are peaks at a value of 2θ, namely 18.78, 31.728, 35.62, 45.47, 53.66, 56.49, 62.81, and 75.31.

XRD analysis also proved that the magnetite compound was formed in-situ in the wood, using strong and weak bases as precursors. In the diffractogram (Figure 4), magnetic jabon wood made with a strong base precursor has peaks at a value of 2θ, namely 32.57, 35.41, 53.67, 57.39, 62.89, and 74.21. In the diffractogram of the magnetic jabon wood created with weak base precursors, there are peaks at a value of 2θ, namely 18.78, 31.728, 35.62, 45.47, 53.66, 56.49, 62.81, and 75.31.

Based on the XRD pattern, the average diameter (D) of Fe_3_O_4_ nanoparticle crystals in magnetic wood can be calculated using the Scherrer Equation (7) [31]:

(7) $$D=\frac{K\lambda }{\beta \cos\theta }$$

where K is the Scherrer constant, λ is the symbol for the X-ray wavelength, β is the full width of the peak at half maximum (FWHM), and θ is the Bragg diffraction angle. Next, the mean diameter of Fe_3_O_4_(D) nanocrystals in magnetic wood was calculated at the most intense peak (311); at these peaks, the size of the magnetite crystal was 10.8642 nm for a strong base and 10.1996 nm for a weak base.

#### 3.2.4. Magnetic Characterisation

**Figure 5:**
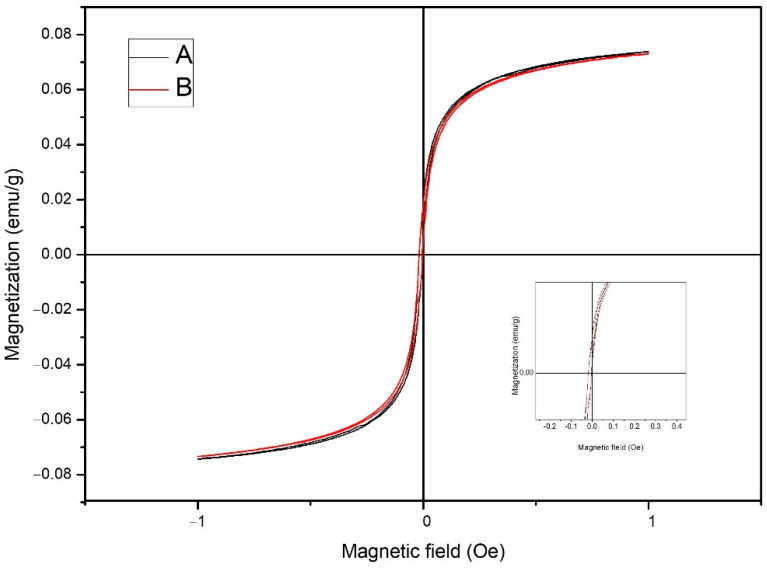
Hysteresis curves (**A**) of jabon wood with a magnetic charge created in-situ with a strong base precursor (black line) and (**B**) magnetically charged jabon wood created in-situ with a weak base precursor (red line).

Magnetic hysteresis curves of magnetic jabon wood samples, synthesized with a strong or weak base, are shown in Figure 5. The hysteresis loop generated from magnetic jabon wood samples has an elongated and narrow shape. Figure 5 shows that magnetic jabon wood synthesized in-situ with either a strong or weak base precursor is classified as a superparamagnetic material with soft magnetic properties.

**Figure 6:**
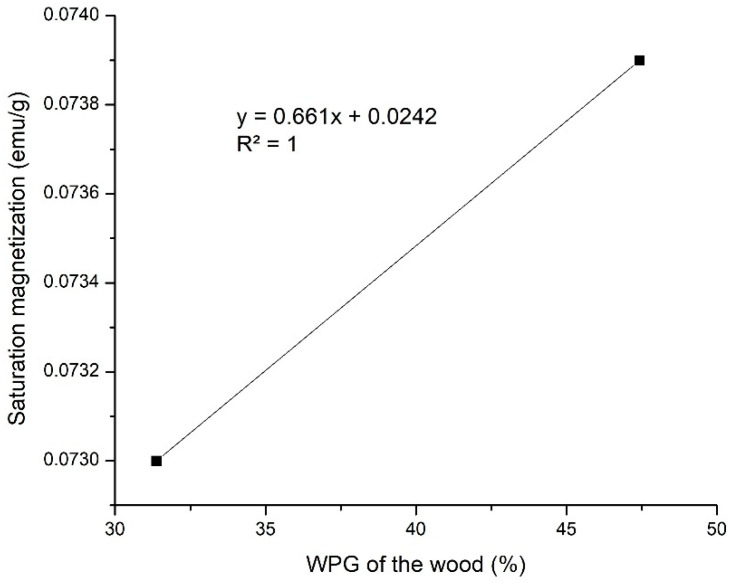
Correlation between weight percent gain (WPG) and saturation magnetisation (Ms) of treated jabon wood.

WPG values were linearly correlated to Ms values (Figure 6). The higher the WPG values, the higher the Ms values; this finding was in line with Gao et al. [32]. The saturation magnetisation (Ms), coercivity (Hc), and retentivity (Mr) of treated jabon wood are shown in detail in Table 3. The M_s_ values for jabon wood treated with the in-situ method using strong and weak base precursors were 0.0739 emu/g and 0.0730 emu/g, respectively. Magnetically charged jabon wood created with a strong base precursor had a higher M_s_ value. The H_c_ value for jabon wood with magnetic charge created in-situ with strong and weak base precursors was 5.97 × 10^−5^ O_e_ and 1.65 × 10^−4^ O_e_; this shows that the magnetically charged wood is classified as having weak magnetic properties because those values are lower than the theoretical value of the bulk magnetite, approximately 60 emu/g for NaOH and 78 emu/g for samples obtained from NH_4_OH in the same condition [23]; this outcome corresponded with the results of Dong et al. [20], who discovered the influence of the wood structure on the magnetic properties of magnetite particles which formed by an in-situ method. Based on Bowyer et al. [33], cell walls mainly consist of cellulose, which is a diamagnetic material [34]. It could cover magnetic nanoparticles, which could lead to a decrease magnetic surface. The magnetic surface is linearly correlated with Ms values [32].

The M_r_ value for jabon wood with in-situ magnetic charge through treatment with strong and weak base precursors was 0.0198 emu/g and 0.0176 emu/g, respectively. These results also show that the M_r_ value of remanent magnetisation in magnetically charged wood is greater in association with strong base precursors compared with weak base precursors. The greater the value of M_r_, the greater the magnetic properties. It is well known that the magnetic properties of Fe_3_O_4_ nanoparticles are affected by the size, shape, and dimensions of the particles. Furthermore, the VSM results revealed that the Fe_3_O_4_ had super paramagnetic behaviours at room temperature; it can be seen from the Hc value, which was close to zero, and the crystallite size of Fe_3_O_4_ which was under 50 nm [35].

## 4. Discussion

### 4.1. Dimensional Stability of Magnetic Jabon Wood

In the in-situ method of magnetite production in wood, the hydroxy ion of the base compound played an important role as the main precursor because the amount of iron (II) and iron (III) ions are reacted at the same concentration. According to Chang [36], the law of reaction equilibrium states that a chemical reaction depends on the reaction equilibrium mechanism. A reaction will shift towards the product (Fe_3_O_4_) if the precursor concentration is higher. NaOH is a strong base that can completely dissociate into sodium ions and hydroxy metal ions, while NH_4_OH is a weak base that does not completely dissociate (Kb = 1.8 × 10^−5^) into ammonium and hydroxy ions [37].

The increased density values of treated wood in the current study were caused by the penetration of the Fe solution into wood cell walls, which resulted in increased weight. Impregnation using a polymer causes the polymer to enter the cell wall and form bonds with the cell wall components [38,39]. When Fe solution is used, cell wall swelling is caused by the Fe solution entering and filling the microvoids in the wood cell wall. As the WPG value increases, the wood density also increases; this is in line with Bowyer et al. [33] who stated that the higher the wood density value, the more wood substance in the cell wall. With polymerisation, the polymer content in wood cell walls makes them thicker; however, the densities of untreated and magnetic jabon wood produced with NaOH and NH_4_OH were not statistically significantly different because magnetite did not form bonds with cell wall components (bulking) (Figure 1 and Figure 2).

Our results contrasted with those from previous studies [21,40,41,42]. The ASE value of magnetic jabon wood with the strong base NaOH (64.3%) used in the in-situ method was lower than with the weak base NH_4_OH (87.0%). Magnetic jabon wood with the NaOH precursor had a high WPG value but a lower ASE value and a higher WU value than that with the NH_4_OH precursor; this outcome was due to the nature of the sodium metal residue from the strong base NaOH. According to Yaws [43], sodium is an alkali metal that easily reacts with water by binding water molecules from its surroundings; this causes magnetic wood with the NaOH precursor to have a lower ASE and a higher WU. According to Daoush [25], the use of a strong base must be adjusted to a mole ratio based on the chemical equation for the formation of magnetite to avoid having an excess base. The excess base can catalyse the degradation of the wood structural unit and thus lead to the degradation of magnetic wood physical properties.

### 4.2. Magnetic Jabon Wood Characterisation

#### 4.2.1. FTIR Analysis

In magnetic jabon wood, Fe-O functional groups were identified and indicated the bonds in Fe_3_O_4_ compounds. Hydroxy groups were also identified both inside and outside the plane in the form of bending vibrations, likely because the magnetite compound can adsorb water molecules on its surface [27]. In magnetic jabon wood synthesised by in-situ reaction with a strong base precursor NaOH or a weak base NH_4_OH, magnetite can be identified by the presence of a functional group of Fe(III)-O, which is the bond formed in Fe_3_O_4_. From the spectrum (Figure 1), we can also see a higher absorption rate in association with the NaOH treatment, with wave numbers indicating the presence of the Fe-O functional group; this finding can indicate that more Fe has entered, and it strengthens the results from testing the physical properties of the wood; it also proves that magnetite has already formed in the wood. In addition, functional groups were identified based on C-H stretching from cellulose and hemicellulose, C-H bending in the aromatic lignin framework, O-H bending in cellulose, and C=O stretching from hemicellulose [44].

#### 4.2.2. Macro and Microscopic Analysis (SEM and SEM-EDX Analysis)

During the in-situ treatment method, the acidic conditions of the iron and ferric chloride solutions and the alkaline conditions of both the NH_4_OH and NaOH solutions caused the wood to become hygroscopic and brittle, leading to the degradation of some of the wood components; this outcome is in accordance with the research of Dong et al. [21]. The measured Fe content reflects the Fe content of the magnetite compounds formed in-situ in the wood, and it is closely correlated to the WPG value of jabon wood; this value was higher when the precursor was a strong base compared with a weak base (Table 1).

#### 4.2.3. XRD Analysis

Based on a comparative analysis of the standard JCPDS (Joint Committee on Powder Diffraction Standard) No. 03-0226, we concluded that the peaks (Figure 3) were cellulose. Cellulose can still be detected in magnetite wood, but the degree of crystallinity cannot be determined due to the very strong magnetite compound. In addition, cellulose can be hydrolysed by bases during the immersion process. With the weak base precursor, there was no detectable value of 2θ at 34.61; this may have happened because the diffraction signal in the crystal lattice was very weak due to the influence of a strong sample matrix background [45]. According to Dong et al. [21], part of the crystalline structure in damaged wood is caused by magnetic treatment.

Based on a comparative analysis of the standard JCPDS magnetic diffractogram No. 19-0629 [46], we concluded that the peaks were magnetite compounds that were successfully formed in jabon wood in-situ. In a comparison of the results, the wood treated with the strong base precursor had more peaks according to the standard magnetite diffractogram. With the strong base precursor, no 2θ values were detected at 18.78 and 45.47 with Miller index values of 111 and 400, respectively; this may have happened because the diffraction signal in the crystal lattice was very weak due to the influence of a strong sample matrix background [45]; this crystal size is smaller than previously reported results [21], with the weak base being associated with a size of 16 nm. According to Sumadiyasa et al. [47], the measurement results using the Scherrer equation represent the crystal size of a certain phase only (single phase); in our case, it was the magnetite phase.

#### 4.2.4. Magnetic Characterisation

According to Tebriani [48], when an object is subjected to a magnetic field or when the magnetic field is removed, the hysteresis curve has a reverse order that is almost symmetrical and also seen from the narrow area of the hysteresis curve. The area of the hysteresis curve shows the energy required for magnetisation. Soft magnets require very little energy for magnetisation; this was confirmed by Tang and Fu [49], who stated that the loop (Figure 5) indicates a magnetic wood sample that exhibits paramagnetic behaviour; however, it is possible that magnetic wood samples also exhibit superparamagnetic behaviour, which is characterised by low retentivity (M_r_) and coercivity (Hc) values [47,48]. According to Tebriani [48], these superparamagnetic properties arise in small materials (1–10 nm). The crystal test with XRD showed that the particle size was 10 nm.

Magnetically charged jabon wood treated with a strong base precursor had a higher M_s_ value in the current study; this finding indicated that the particle size of the magnetite created with a strong base precursor is larger. Further, according to Bukit et al. [50], the larger the particle size of the magnetite, the greater the number of magnetic domains and the larger the boundary of the magnetic domain, which leads to an increase in the saturation magnetisation value (M_s_).

Based on H_c_ data, the magnetically charged wood had a smaller H_c_ value if a strong base precursor was used compared with a weak base precursor; this shows that the particle size of magnetite created with an in-situ process is larger if a strong base is used rather than a weak base. According to Bukit et al. [50], the H_c_ value is inversely proportional to the size of the magnetite particles formed in wood; that is, the larger the particle size, the smaller the H_c_ value and vice versa. A smaller magnetite particle size means there are more boundaries between the crystals and a greater barrier to the wall motion domain. As a result, the resistance to the demagnetising field increases, which means the H_c_ value is higher. Conversely, the larger the magnetite particle size, the easier it is to move the domain walls; therefore, the resistance to the demagnetising magnetic field becomes smaller and the Hc value is lower.

The M_s_, M_r_, and H_c_ values of magnetic wood samples were lower than those reported in a previous study [20]. The low values of M_s_, M_r_, and H_c_ were likely caused by the low percentage of magnetic particles formed during the coprecipitation process in wood. The more nano Fe_3_O_4_ formed and deposited in the cell walls of the wood, the more the magnetic properties of the wood are increased [21,49]. According to Merk et al. [17], the longitudinal direction of the magnetized wood is better than the tangential and radial directions in that more nano Fe_3_O_4_ is distributed into the wood cell walls; this causes the magnetic charge of the wood to be higher along the longitudinal axis, and was apparent from the SEM results in our study, which showed a fairly thick layer of iron magnetite on the pore walls.

## 5. Conclusions

Based on our results, we are able to conclude the following:

1. The density and BE were increased in magnetic jabon wood, while WU was decreased for both precursors. ASE of magnetic jabon wood was 64.5% with the NaOH precursor and 87.1% with the NH_4_OH precursor.
2. Based on FTIR analyses, magnetite was successfully synthesised in jabon wood; further, based on SEM-EDX analysis, the iron magnetite particles stuck to the cell walls of jabon wood with a value of 40% for NaOH precursors and 23% for NH_4_OH precursors.
3. XRD tests showed that the crystal sizes of Fe_3_O_4_ obtained from syntheses with strong and weak bases as precursors were almost the same, namely, 10.8642 nm and 10.1996 nm. The crystals formed were Fe_3_O_4_ crystals.
4. VSM analyses showed that magnetic jabon wood synthesized in-situ with strong and weak base precursors was classified as a superparamagnetic material with soft magnetic properties.
5. Magnetically charged jabon wood was successfully synthesised in-situ with strong and weak base precursors, but based on the results of physical properties and characterisation tests, the in-situ method using a strong base had better results. The use of a strong base must be adjusted to the mole ratio according to the chemical equation for the formation of magnetite so that there is no excess base that can catalyse the degradation of the wood structural unit.

## Figures and Tables

**Table 1:** Data on the dimensional stability of magnetic jabon wood.

Treatment	Oven-Dried Density	WPG	BE	WU	ASE
Untreated	0.32 ± 0.04 ^a^	0.00 ^a^	2.21 ± 1.00 ^a^	125.17 ± 16.62 ^a^	-
In-situ NaOH	0.36 ± 0.01 ^a^	47.44 ± 7.85 ^b^	7.13 ± 2.81 ^b^	110.89 ± 12.81 ^a^	64.26 ± 9.76 ^b^
In-situ NH_4_OH	0.34 ± 0.04 ^a^	31.38 ± 4.99 ^c^	5.88 ± 1.13 ^b^	104.38 ± 12.43 ^a^	87.05 ± 14.63 ^c^

^a^^–c^ Values followed by different letters are significantly different based on the Duncan test.

**Table 2:** SEM-EDX test results.

Sample	Fe (% Wt)
In-situ NaOH	40.11
In-situ NH_4_OH	23.39

**Table 3:** The saturation magnetisation (M_s_), coercivity (H_c_), and retentivity (M_r_) of the treated jabon wood.

Sample	M_s_ (emu/g)	M_r_ (emu/g)	H_c_ (O_e_)
In-situ NaOH	0.0739	0.0198	5.97 × 10^−5^
In-situ NH_4_OH	0.0730	0.0176	1.65 × 10^−4^

## Data Availability

Not applicable.
